# Mechanical Alloying: An Advantageous Method for the Development of Mg_2_Si_0.8_Sn_0.2_ and Mg_2_Si Thermoelectrics Using Commercial and Recyclable Silicon

**DOI:** 10.1021/acsaem.4c03000

**Published:** 2025-01-22

**Authors:** Panagiotis Mangelis, Panagiotis S. Ioannou, Anne-Karin So̷iland, Theodora Kyratsi

**Affiliations:** † Department of Mechanical and Manufacturing Engineering, University of Cyprus, 1678 Nicosia, Cyprus; ‡ ReSiTec AS, Setesdalsveien 110, 4617 Kristiansand, Norway

**Keywords:** magnesium silicide, mechanical alloying, solid-state reaction, hot-press sintering, recycled silicon, thermoelectric properties

## Abstract

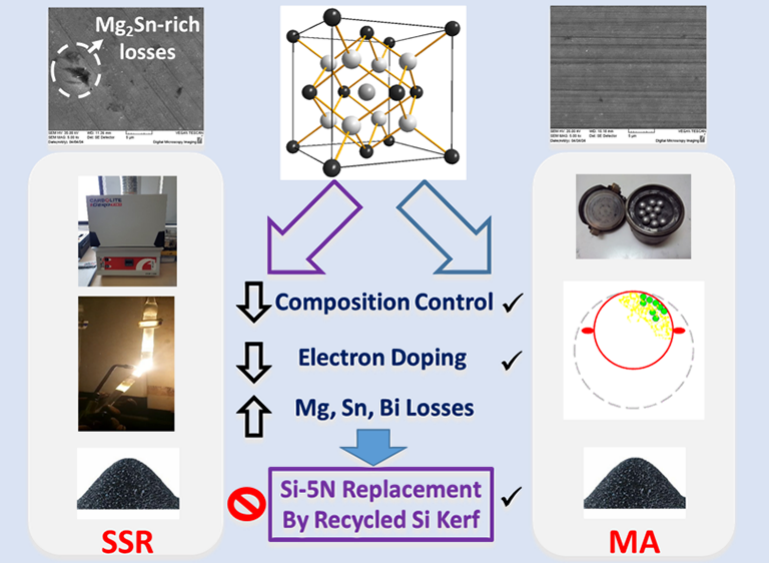

A comparative study of Bi-doped Si-rich silicide phases, Mg_2_Si_0.8_Sn_0.2_ and Mg_2_Si, is reported, investigating in parallel two different synthetic routes: the solid-state reaction (SSR) and mechanical alloying (MA). Both synthetic routes produce the desired silicide phases. However, powder XRD Rietveld refinements reveal appreciable Mg and Sn losses for the SSR-developed Mg_2_Si_0.8_Sn_2_, while EDS measurements also confirm Sn losses together with a decrease in the Bi content. This has a strong impact in electrical transport properties, indicating a severe electron doping deficiency. In contrast, the EDS results for MA-based phases are in a good agreement with the nominal values, indicating an effective Bi doping. Moreover, considering the Rietveld refinement results and SEM analysis, notable changes in the content of Mg interstitial atoms at the 4*b* crystallographic site seem to be correlated with the microstructure features of the two MA compounds. Electrical conductivity and Seebeck coefficient measurements confirm the aforementioned results. In addition, a small reduction in lattice thermal conductivity is observed for the two MA systems due to the nanostructuring effect. At 773 K, *ZT* values of 0.85 and 0.6 are exhibited for Mg_2_Si_0.8_Sn_0.2_ and Mg_2_Si, respectively. MA is proven to be an advantageous route for the development of Si-rich phases since it provides a better control of doping and higher precision of produced stoichiometric compositions, while in parallel it is a straightforward and scalable method. The replacement of commercial Si by two types of recycled Si-kerf is also attempted here. The kerf-based materials exhibit small reductions in *ZT*, giving prominence to the efforts to utilize more effectively recyclable Si.

## Introduction

Nowadays, the growing needs for energy consumption and, in addition, the environmental demands for the decrease of carbon emissions globally make ever more urgent the development of new and environmentally friendly energy technologies. Thermoelectric (TE) generators have the potential to provide considerable energy savings since vast amounts of energy globally are lost to the environment as waste heat through industrial processes.^1^ Practically, the low performance and high cost of the current TE materials do not allow mass-production and large-scale applications, limiting them to very specific applications where other factors such as reliability are of high importance.^2,3^ However, recent advancements of TE materials grow the expectations that in the following years, new TE devices will be developed with high efficiencies and lower costs made of earth-abundant materials, allowing TE technology to become more competitive in the green energy market.^4^

Various groups of materials, such as GeTe,^5^ Bi_2_Te_3_,^6^ PbTe,^7,8^ and half-Heusler compounds,^9^ are being explored for power generation applications, demonstrating quite promising TE properties. The performance of TE materials is dependent on the electrical conductivity (σ), the Seebeck coefficient (*S*), the absolute temperature (*T*), and the thermal conductivity (κ). These properties are included in the formula of a dimensionless figure of merit, *ZT* = *S*^2^σ*T*/κ, which determines the TE efficiency of materials.^3^ Current commercially available TE modules made of telluride alloys exhibit *ZT* values close to 1. However, the high cost and toxicity of Te are forbidden for large-scale production and applications.^10−12^ Intensive research efforts are currently carried out to discover Te-free alternatives, low-cost, environmentally friendly, and nontoxic materials with high TE performance^13,14^ and to solve several practical issues, so that new TE generators will be able for mass-production and commercialization in the future.^15^

Silicide compounds with the general formula Mg_2_Si_1–*x*_Sn_*x*_ are low-cost, earth-abundant, nontoxic materials with promising TE efficiencies reaching remarkable *ZT* values between 1 and 1.7.^16−23^ Mg_2_Si-based solutions are crystallized in the antifluorite cubic crystal structure with space group *Fm*-3*m*. Si^4–^ ions occupy the 4*a* (0 0 0) site, while the internal positions at the 8*c* (1/4 1/4 1/4) site are occupied by Mg^2+^ ions.^24−26^ Bismuth and antimony have been proven to be the most efficient electron dopants for tuning and optimization of the electrical transport properties of these materials. Substituting silicon at the 4*a* site and donating one electron, significant increases in electron density and electrical conductivity have been achieved. Small amounts of Bi or Sb substitution close to 2 and 3 at % have shown great improvements in electrical transport properties and the TE efficiency.^18−20,23,24^ Substitution of Mg at the 8*c* (1/4 1/4 1/4) by Cr and Al dopants has also presented promising properties,^27−30^ while implementing double substitution with Bi and Cr in Mg_2_(Si_0.3_Sn_0.7_) a record high *ZT* of 1.7 has been exhibited.^21^ Moreover, several reports have revealed the great importance of Mg excess to the electron doping of silicide phases since a fraction of Mg excess occupies the interstitial 4*b* site, donating two electrons and becoming Mg^2+^.^31−34^ Mg interstitial atoms (Mg_*i*_) can work together with Bi and Sb dopants, achieving notable improvements of carrier concentration and electrical conductivity and providing in parallel an alternative route to control electron doping for tuning the electrical transport properties and optimizing the TE efficiency of silicide compounds.^24,32^

Most of the investigations of Mg_2_(Si, Sn) solid solutions have been focused mainly on the Mg_2_Si_0.6_Sn_0.4_ and Mg_2_Si_0.4_Sn_0.6_ phases since these systems have shown the best TE efficiencies, while in parallel the binary Mg_2_Si has also been studied extensively due to its structural simplicity. Very few studies have been reported for Si-rich solutions such as Mg_2_Si_0.8_Sn_0.2_.^35−37^ Although Si-rich solutions demonstrate lower TE efficiencies compared with their Sn-rich counterparts, they exhibit other important beneficial characteristics, such as lower densities as well as better thermal and chemical stability, which allow for a wider range of TE applications. Recently, de Boor et al. reported the development of Sb-doped Mg_2_Si_0.8_Sn_0.2_ compounds by direct melting, reaching a maximum *ZT* of 0.95 at 740 K, while Bi-doped phases prepared by solid-state reaction (SSR) and spark plasma sintering (SPS) exhibited a *ZT* of 1.17 at 850 K.

It has been proven that the material development by different synthetic methods significantly influences the structure as well as their physical and chemical properties. Our previous study presented the beneficial results for mechanical alloying (MA) in comparison with the SSR method for the development of Mg_2_Si_0.55-x_Sn_0.4_Ge_*x*_ (x = 0 and 0.05) phases, since it is a simple, straightforward, and scalable synthetic route.^18^ The avoidance of high-temperature treatments results in lower losses due to Mg evaporation and thus a better control of Mg content. Therefore, its incorporation in the structure as Mg_*i*_ may contribute more effectively to the electron doping and the improvement of electrical conductivity. Moreover, the nanostructuring effect induced by MA leads to a significant reduction of lattice thermal conductivity.^18,38,39^

Few studies have been focused on the development of Si-rich silicide phases by MA since several problems arise during this synthetic method. Brittle materials with high bulk modulus are ideal for ball milling since they facilitate a continuous fracturing and welding process without aggregation problems.^38^ On the other hand, Mg is a ductile and soft element, which makes the ball milling process difficult due to its aggregation in the vial. The first efforts to synthesize Mg_2_Si by MA were carried out by Li and Kong using a QF-I planetary mill.^40^ However, the formation of the desired silicide phase was not achieved even for a milling time of 100 h. The introduction of organic additives together with the reagent elements was attempted by Niu and Lu using a planetary ball mill in order to facilitate the milling process and prevent Mg agglomeration.^41^ However, after milling for 30 h, the formation of considerable amounts of secondary phases such as magnesium oxides and hydrides is unavoidable. Bux et al. finally managed to develop a single-phase of Mg_2_Si, applying an incremental milling technique where small amounts of Mg were added to the vial to react slowly with silicon powder.^42^

Here, we report a more simple and straightforward ball milling route for the Si-rich silicide compounds, where Bi-doped Mg_2_Si_0.8_Sn_0.2_ and Mg_2_Si phases were developed for the first time exclusively by the MA method, initializing the synthesis process with the introduction of all stoichiometric amounts of reagent elements. In addition, a comparative study of MA and SSR synthetic routes is presented for the two Si-rich systems based on structural investigations, chemical analysis, and TE property measurements. It is validated that MA provides a better control and higher accuracy in the stoichiometric composition of final products, favoring in parallel Bi substitution, which results in a more effective electron doping. Subsequently, in an effort to utilize more effectively recyclable byproducts of industrial processes and expand our research on the development of silicide thermoelectrics by using recycled Si,^43−45^ two types of kerf from the photovoltaic (PV) industry are used for the development of Si-rich phases following the MA method, which is proven in this study to be an advantageous synthetic route compared with the SSR. The replacement of commercial Si by recyclable Si kerf for the development of high-performance TE materials is of great importance, since it provides an effective waste management solution in the silicon industry, achieving in parallel a circular economy approach in the green energy sector.^46,47^

## Experimental Section

Bi-doped Mg_2_Si_1–*x*_Sn_*x*_ (*x* = 0 and 0.2) were synthesized by MA following the experimental conditions of the previous report.^18^ Bi doping of 3 at % by substitution of Si was selected since our previous studies have shown the best results for the specific composition.^18,24^ Appropriate stoichiometric amounts of reagent elements with a purity greater than 99.8% from Alfa Aesar were mixed and sealed in a tungsten carbide vial under an argon atmosphere with a ball-to-powder ratio of 10:1. MA was carried out at 400 rpm for 40 h in a planetary mill (Pulverisette 6, Fritsch) with intermediate pauses every 5 min in order to avoid heating and Mg evaporation as well as interruptions every 8 h to strip and regrind the agglomerated powder from the vial wall. Excess of 10 at % Mg was added to the nominal compositions to compensate for the Mg loss during synthesis.^18^

For the SSR synthesis of Bi-doped Mg_2_Si_0.8_Sn_0.2_, the material was prepared according to the process of the previous study combining short-time ball milling and heat treatment.^24^ The results of 3 at % Bi-doped Mg_2_Si from the previous report are also included in this study for comparison, referred to as SSR Mg_2_Si.^24^ Appropriate stoichiometric amounts of starting elements were mixed in a glovebox under an argon atmosphere and sealed in a tungsten carbide vial with a ball-to-powder ratio of 23:1. Ball milling was carried out at 300 rpm for 60 min with intermediate interruptions of 5 min every 15 min to avoid heating. The resulting powder was cold pressed at 0.5 GPa into pellets in order to ensure good contact between the grains and enhance the diffusion mechanism. The pellets were placed inside graphite crucibles and sealed into silica tubes under a high vacuum. Then, they were heated at temperatures of 400 and 600 °C for 1 h.

The densification of developed powders into pellets was carried out uniaxially through hot-press sintering under an argon atmosphere at 820 and 860 °C for Mg_2_Si_0.8_Sn_0.2_ and Mg_2_Si, respectively, with a pressure of 80 MPa for 1h in a HP20, Thermal Technologies system.

Powder X-ray diffraction (XRD) measurements were carried out using a Rigaku SmartLab diffractometer, which operates with a Cu–Kα source at 9 KW (45 kV, 200 mA). A scan time of 0.6 s per step and a scan step of 0.02° over the angular range 10 ≤ 2θ/° ≤ 90 were set. The Rietveld method was performed by using the General Structure Analysis System (GSAS) software package.

Scanning electron microscopy (SEM) was used for the microstructure characterization of investigated materials. Hot-press sintered pellets were examined using a backscattered electron (BSE) detector. SEM imaging was performed using a Tescan Vega II LSU, a thermionic emission electron microscope, at an accelerating voltage of 20 kV. Energy-dispersive X-ray spectroscopy (EDS) was employed for the elemental analysis of investigated samples using the Vega II LSU, equipped with a Princeton Gamma Tech EDS detector. Multiple scans with a beam energy of 20 kV, a live time of 100 s, and a takeoff angle of 45° were performed on each polished sample, and the corresponding EDS spectra were extracted. Average mass fractions and atomic percentages of the constituent elements were then calculated based on the ZAF matrix correction quantification protocol.

The experimental density of pellets was calculated by a geometrical method. Using a ZEM – 3 ULVAC – RIKO electrical conductivity (σ) and Seebeck coefficient (*S*) measurements were carried out under a helium atmosphere in the temperature range 300 K ≤ *T* ≤ 773 K. The thermal diffusivity (*D*) and specific heat capacity (*C*_p_) of the samples were measured by a Netzsch LFA 457 laser setup. Data were collected in 50 K increments on pellets coated with graphite. A pyroceramic reference sample was used for *C*_p_ measurements. The thermal conductivity was determined by using the formula: κ = *D*ρ*C*_p_. Estimated uncertainties for the measurements of electrical and thermal transport properties are ±5% and ±10%, respectively.

## Results and Discussion

Bi-doped Mg_2_Si_0.8_Sn_0.2_ is developed by two different synthetic methods, MA and SSR, and Bi-doped Mg_2_Si is also produced by MA, which is compared with its SSR counterpart synthesized in the previous study.^24^ Structural and morphological studies were performed on all investigated samples. The effectiveness of the two synthetic techniques is investigated comparably in terms of electrical and thermal transport properties in both systems. For this reason, the standard substitution of Si by Bi of 3% for all investigated materials is presented. Moreover, depending on the synthesis method, certain deviations from nominal compositions are observed and discussed. The replacement of pure Si by two types of kerf in both systems is also investigated for the case of the most promising synthetic route.

### Synthesis and Material Characterization of Bi-Doped Mg_2_Si_0.8_Sn_0.2_ and Mg_2_Si

**Figure 1:**
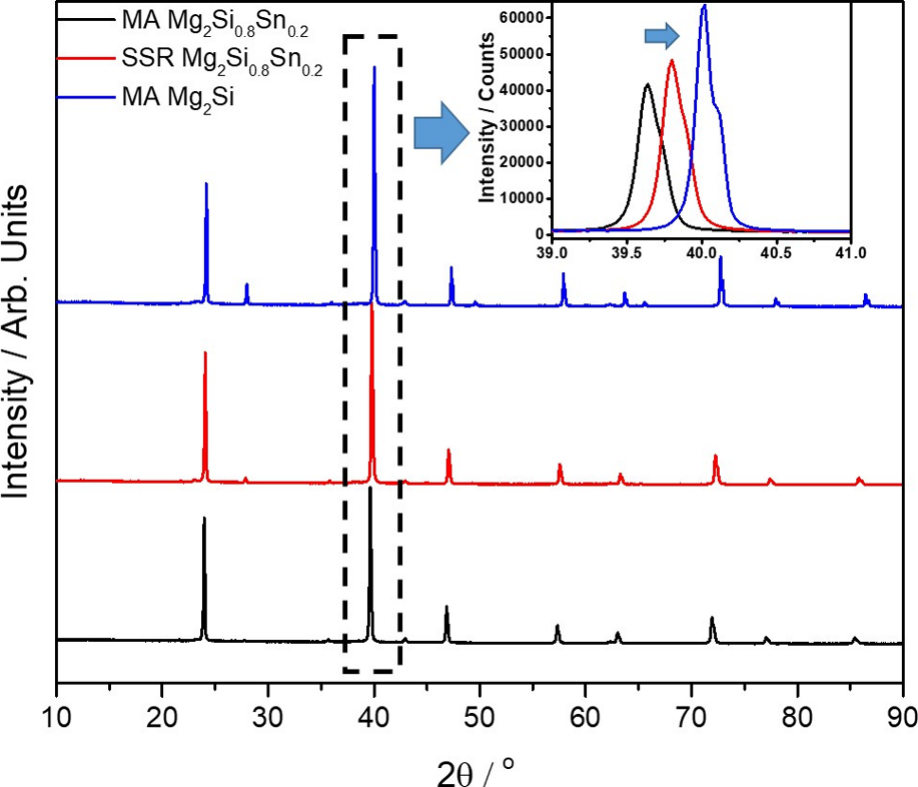
Powder XRD data in arbitrary units for the MA- and SSR-based Mg_2_Si_0.8_Sn_0.2_ as well as the MA-based Mg_2_Si in the angle range of 10 ≤ 2θ/° ≤ 90. Inset: The (220) peak intensity in real counts for the three investigated phases. The arrow indicates the (220) shift of SSR-based Mg_2_Si_0.8_Sn_0.2_ to a higher angle position compared with that of its MA-based counterpart.

During the MA process, in order to avoid the aggregation problems of Mg due to its high ductility, every 8 h of ball milling, the agglomerated material was removed from the vial with regrinding in the glovebox. A ball milling of 40 h was required for the synthesis of Mg_2_Si_0.8_Sn_0.2_ and Mg_2_Si. Compared with the Sn-rich phases,^44^ 8 h more of ball milling was necessary for the formation of the desired Si-rich silicide phases since Si needs higher impact energy and thus, longer time to react with Mg due to its higher moduli values than those of Sn. Powder XRD measurements were performed to investigate the purity of the produced materials and the existence of possible secondary phases. Figure 1 shows the XRD patterns of the two MA-developed products attributed to the stoichiometry of Mg_2_Si_0.8_Sn_0.2_ and Mg_2_Si, as well as that of the Mg_2_Si_0.8_Sn_0.2_ compound synthesized by the SSR method. All investigated materials present high purity, forming the desired silicide phases, apart from traces of MgO, which are detected at low levels. What is notable here from the qualitative analysis is that a systematic shift of all peaks is observed for the SSR-based phase to higher angles, getting closer to those of the Mg_2_Si phase, in comparison with the MA-based counterpart (Figure 1, inset). This indicates structural changes in the unit cell that may be related to stoichiometric deviations of two compounds.

**Figure 2:**
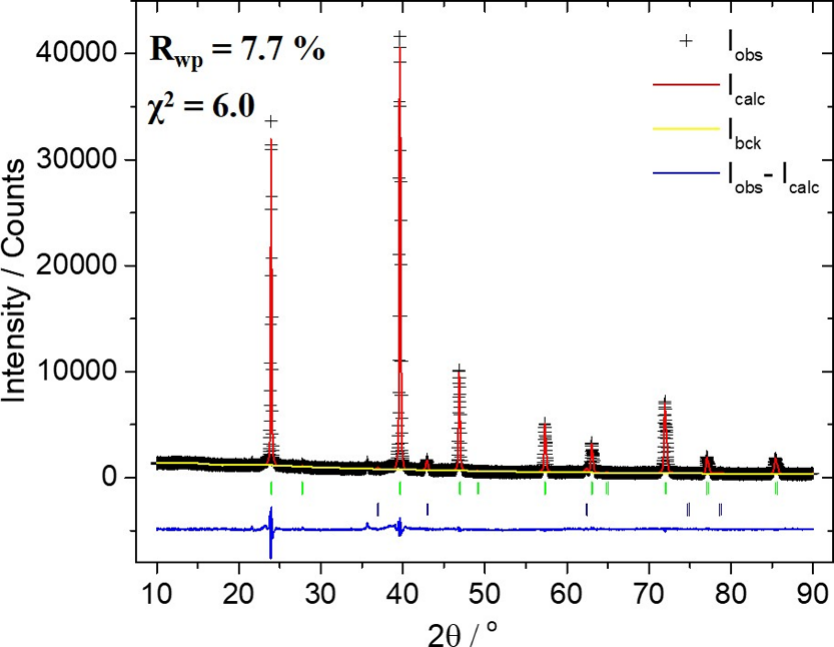
Powder XRD Rietveld refinement profile for the MA-based Mg_2_Si_0.8_Sn_0.2_: final observed (black crosses), calculated (red solid line), calculated background (yellow line), and difference (blue line). Reflection positions for the silicide phase are marked with green and for MgO with navy color.

Rietveld refinements were performed for Mg_2_Si and the two Mg_2_Si_0.8_Sn_0.2_ compounds developed by MA and SSR. Figure 2 shows the XRD refinement profile of Mg_2_Si_0.8_Sn_0.2_ developed by MA, while the remaining refinement profiles of SSR-based Mg_2_Si_0.8_Sn_0.2_ and MA-based Mg_2_Si compounds are presented in Supporting Information. The refinements involved smoothly, and the thermal parameters of all elements were constrained to be equivalent. As can be observed, the calculated profile fits well with the experimental data for all investigated materials (7.2 ≤ *R*_wp_/% ≤ 9.1), confirming the antifluorite-type cubic structure with the space group *Fm-*3*m*, where Mg atoms occupy the 8*c* (1/4 1/4 1/4) crystallographic site and Si/Sn/Bi occupies the 4*a* (0 0 0) site. Small percentages of MgO were also confirmed for all developed materials. MgO is a quite common secondary phase also identified in several previous studies.^19,24,36,48^

Taking into account the difference in the angle positions observed previously for the peaks of MA- and SSR-based Mg_2_Si_0.8_Sn_0.2_ compounds, refinements on the occupancy factors at the two crystallographic sites were attempted. In order to avoid correlation problems, a constraint was applied at the 4*a* site to keep the site fully occupied. The refinement of the SSR-based sample reveals a remarkable reduction of Sn percentage, improving in parallel the refinement profile with a reduction of *R*_wp_ value from 7.8 to 7.2%. In contrast, the refined occupancies of Si and Sn for the MA-based compound remained very close to the nominal values without any improvement in the refinement profile (*R*_wp_ = 7.7%). Refining the occupancy of Mg at the 8*c* site, there was also no change from the nominal value for both systems. For this reason, the site occupancy factors of these atoms were fixed at the nominal compositions since no changes were observed by refining them. Table 1 presents the results from the Rietveld refinements. The refined lattice constant value of the SSR-based phase shows a reduction compared to the MA-based counterpart, justifying the peak shift to higher angles. This also agrees with the refined results of the Si/Sn occupancies of the two systems. The reduction in lattice constant with the increase of Si content is an anticipated result since the Si atom is smaller than Sn. This results in a decrease of unit cell volume of *ca*. 1.4% and *ca*. 3% for the SSR-based Mg_2_Si_0.8_Sn_0.2_ and the binary Mg_2_Si, respectively.

**Table 1:** Refined Parameters from Rietveld Analysis of Powder XRD Data for Bi-doped Mg_2_Si_1–*x*_Sn_*x*_, (*x* = 0 and 0.2), Developed by SSR and MA Methods

Si-rich phases	*a* [Å]	*V* [Å^3^]	Mg_*i*_^a^ [%]	Si^b^ [%]	Sn^b^ [%]	*U*_iso_ [Å^2^]	MgO [wt %]	*R*_wp_ [%]
Mg_2_Si_0.8_Sn_0.2_								
SSR	6.39359(3)	261.357(4)	0.1(1)	89.4(2)	7.6(2)	1.42(3)	2.6(1)	7.2
MA	6.42402(4)	265.106(5)	8.4(2)	77.0	20.0	1.47(4)	5.3(1)	7.7
Mg_2_Si								
MA	6.35876(3)	257.109(3)	1.2(3)	97.0		1.04(4)	4.1(1)	9.1

a Occupancy of interstitial Mg at the crystallographic site 4*b* (1/2 1/2 1/2).

b Occupancy at the crystallographic site 4*a* (0 0 0).

The occupancy of Mg atoms (Mg_*i*_) at the interstitial site 4*b* (1/2 1/2 1/2) was also investigated through Rietveld analysis since Mg excess of 10 at. % was added for all investigated samples. As can be observed from Table 1, the MA-based Mg_2_Si_0.8_Sn_0.2_ phase presents a percentage of Mg_*i*_ atoms of 8.4%, while for the SSR-based counterpart and Mg_2_Si phase, there is a clear reduction at lower levels. This looks quite strange, and further investigation is required. Although the decrease of Mg_*i*_ content is abrupt for both compounds, the two cases must be examined separately. The following analysis shows that different reasons seem to be responsible for the aforementioned results.

**Figure 3:**
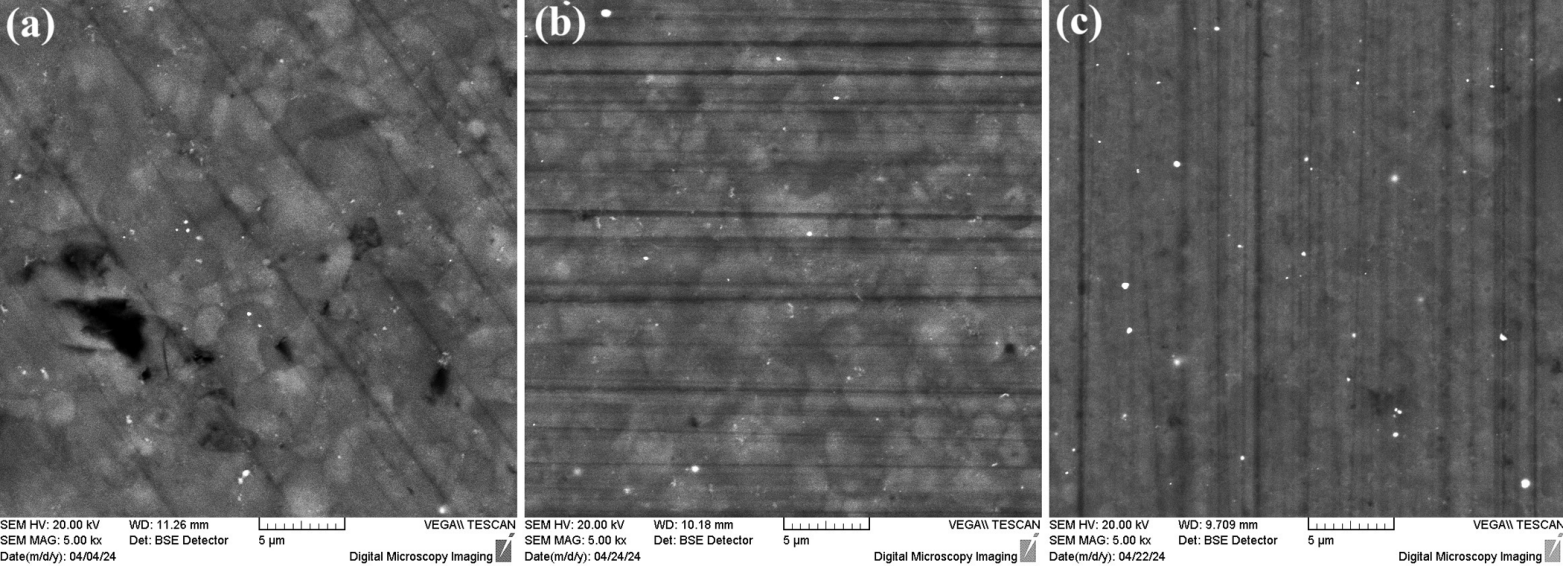
Backscattering SEM imaging for the (a) SSR-based Mg_2_Si_0.8_Sn_0.2_, (b) MA-based Mg_2_Si_0.8_Sn_0.2_, and (c) MA-based Mg_2_Si. The scale bar in these images is 5 μm.

Backscattering SEM imaging was performed on the sintered samples in pellet form to study their microstructure (Figure 3). Interestingly, for both developed Mg_2_Si_0.8_Sn_0.2_ cases (Figure 3a,b), multiple phases are observed. The light gray regions represent Sn-rich phases in comparison with the dark gray matrix, which corresponds to phases with higher Si content. Multiple phases have also been confirmed for Mg_2_Si_0.8_Sn_0.2_ in a previous report.^36^ In addition, regions of high porosity are observed in the microstructure of SSR-developed solid solution, indicating material losses that are probably caused during the synthesis process or hot-press sintering. On the other hand, as expected, in Figure 3c no multiple phases are observed for the binary Mg_2_Si phase which presents a homogeneous microstructure. EDS measurements were carried out to identify the experimental stoichiometry of the developed materials. The results from elemental analysis are presented in Table 2. A remarkable reduction of Sn content is revealed for the SSR-developed material compared with its MA counterpart, which is much closer to the nominal value. This strongly confirms the previous results of refined Si/Sn occupancy factors from the Rietveld analysis. From EDS and XRD Rietveld analysis, it is clearly obvious that an appreciable Sn loss occurs during the synthesis and hot-press sintering process of SSR-based Mg_2_Si_0.8_Sn_0.2_. According to the thermal stability study of Yin et al.,^49^ Mg and Sn losses take place in Si/Sn solid solutions due to a peritectic reaction which forms a Mg_2_Sn-rich liquid phase. This phase is unstable and decomposes, causing Mg evaporation at high temperatures. The study also showed that this phenomenon is severe when the thermal treatment occurs under a high vacuum. The brownish color on the quartz tube after heating is indicative of Mg evaporation and reaction with SiO_2_.^49^ In our study, a similar color is observed on the tube after heating in the SSR case. The abrupt reduction of Mg_*i*_ content to zero indicates that the whole Mg excess of 20% is lost together with *ca*. 10% of Sn. This value is in a very good agreement with the average value of Sn content extracted from the results of EDS analysis and the Rietveld refinement (∼10%). The aforementioned striking results for the Mg_2_Sn-rich losses seem to be also validated by the SEM imaging, which reveals a porous structure for the SSR case, indicative of the decomposition and Mg_2_Sn losses.^49^ Moreover, EDS showed a clear reduction of Bi content, about half of the nominal value, for the SSR case compared with those of MA-based materials, which were very close to the value of 0.03. This indicates that almost half of the Bi content is also lost together with the Mg and Sn losses. This may be explained by the fact that Bi with an atomic radius closer to that of Sn probably shows a preference for the Sn-rich phase in the Si/Sn solid solution. Our previous electron channeling study shows that Bi tends to substitute mainly Sn atoms at the Sn-rich phases in Mg_2_Si_1–*x*_Sn_*x*_ solutions, enhancing this suggestion.^25^ Therefore, it may be possible for a rich Sn–Bi alloy to be formed during Mg evaporation with a low melting point, analogous with rich Sn–Sb islands that were also observed after the decomposition and Mg evaporation in the Sb-doped Si/Sn solution.^49^ As shown later, this deficiency in Bi doping will have a strong impact on the electrical transport properties of the SSR-based material.

**Table 2:** Results from the EDS Elemental Analysis of Bi-doped Mg_2_Si_1–*x*_Sn_*x*_, (*x* = 0 and 0.2), Developed by SSR and MA Methods

Si-rich phases	Si	Sn	Bi
Mg_2_Si_0.8_Sn_0.2_			
nominal	0.770	0.200	0.030
SSR	0.863(4)	0.122(3)	0.015(2)
MA	0.806(1)	0.166(2)	0.027(1)
Mg_2_Si			
nominal	0.970		0.030
MA	0.972(1)		0.028(1)

On the other hand, the EDS measurements for the MA-based compounds showed slight deviations of Si, Sn, and Bi content from the nominal values, indicative of no appreciable losses. In addition, SEM imaging did not show a porous surface for both MA phases, confirming the outputs from the EDS and Rietveld analysis. The aforementioned results of the three characterization techniques lead to the conclusion that the MA method does not cause Mg_2_Sn-rich losses. The main reason seems to be the avoidance of heat treatment at high temperatures during the synthesis process. Moreover, it is concluded that no losses occur during the hot-press sintering process for the MA-based solid solution. Although the same hot-press sintering conditions were applied for both Mg_2_Si_0.8_Sn_0.2_ materials, no Mg_2_Sn-rich losses were observed in the MA case. It is suggested here that probably the reduction of grain size, due to the ball milling process, results in the increase of grain boundaries, which may block the aggregation of the Mg_2_Sn-rich phase into wide regions and consequently prevent appreciable losses in the liquid phase at high temperatures. Comparing the backscattering SEM imaging of two developed Mg_2_Si_0.8_Sn_0.2_ phases (Figure 3a,b), it is shown that the number of smaller Mg_2_Sn-rich (light gray) aggregates is increased in the MA case, indicating a reduction of the average grain size due to ball milling. The increase of smaller aggregates (with a size of few hundreds of nanometers) with increasing ball milling time has also been confirmed in our previous study, carrying out SEM analysis during the MA process.^44^ As shown later, this nanostructuring effect in the two MA-based phases seems to be responsible for a favorable reduction in lattice thermal conductivity.

**Figure 4:**
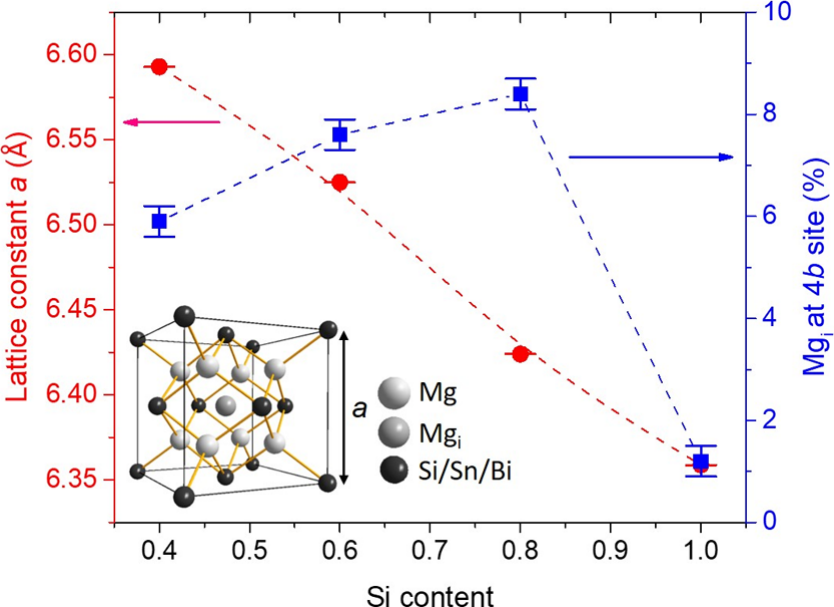
Refined lattice constant and Mg_*i*_ content of 3 at. % Bi-doped Mg_2_Si_*x*_Sn_1–*x*_ (0.4 ≤ *x* ≤ 1) phases developed by MA as a function of Si content derived by powder XRD Rietveld analysis. The refined results for Si content values of x = 0.4 and 0.6 come from our previous report.^44^ Inset: The antifluorite-type cubic structure (space group: *Fm*-3*m*) where Mg_*i*_ atoms occupy the 4*b* (1/2 1/2 1/2) site.

Regarding the notable changes in Mg_*i*_ content between the two MA-developed systems, we cannot conclude whether higher Mg losses occurred during the synthesis process for the binary Mg_2_Si phase, since the same ball milling conditions were applied for both cases, and therefore, the question about the sharp reduction of Mg_*i*_ remains. It must be noted here that a previous study has also shown a low percentage of Mg_*i*_ content for the binary phase. Interestingly, the refined Mg occupancy at the 4*b* site (*ca*. 1.2%) is in a very good agreement with the values of Kubouchi et al.^34^ obtained for the cases of 10–30% Mg excess, validating our refinement results. In an attempt to explore the evolution of Mg_*i*_ content as a function of Mg_2_(Si, Sn) composition, Figure 4 presents the refined lattice constant and Mg_*i*_ content for the MA-based Mg_2_Si_*x*_Sn_1–*x*_ (0.4 ≤ *x* ≤ 1) series as a function of Si composition, incorporating the results of our previous Rietveld analysis.^44^ As can be observed, the refined Mg_*i*_ content of Si-5N Mg_2_Si_0.6_Sn_0.4_ (*ca*. 7.6%) is relatively close (<1%) to the present refined value of (*ca*. 8.4%). The abrupt decrease of Mg_*i*_ content, moving to the Si-end member, seems not to be correlated with a notable change in the lattice constant. The lattice constant is reduced almost linearly as a function of Si content, following Vegard’s law (Figure 4). This is in agreement with previous studies which have shown that Mg_*i*_ content does not cause any effect on the unit cell size because Mg^2+^ exhibits a small ionic radius.^31,33^ Therefore, the sharp Mg_*i*_ reduction may be correlated to the fact that there is no Si/Sn solid solution anymore. It may be possible for Mg_*i*_ atoms to show a preference for the Mg_2_Sn-rich phases in the Si/Sn solution because these regions exhibit locally a larger unit cell compared with the Mg_2_Si-rich ones. This may be favorable for the occupancy of Mg_*i*_ atoms at the central 4*b* site. As backscattering SEM imaging showed, the two Mg_2_Sn- and Mg_2_Si-rich regions in the microstructure of MA-based Mg_2_Si_0.8_Sn_0.2_ are quite distinguishable (and in contrast with the homogeneous structure of the binary phase), supporting this suggestion.

### TE Characterization and Figure-of-Merit of Bi-Doped Mg_2_Si_0.8_Sn_0.2_ and Mg_2_Si

**Figure 5:**
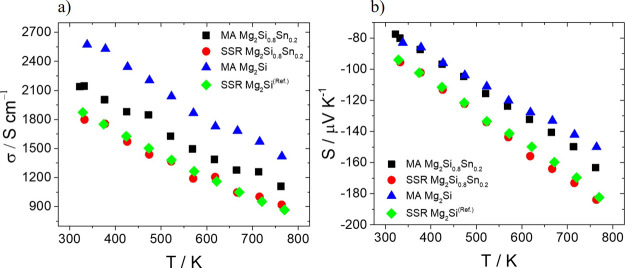
(a) Electrical conductivity and (b) Seebeck coefficient as a function of temperature for Bi-doped Mg_2_Si_0.8_Sn_0.2_ and Mg_2_Si developed by SSR and MA. Data for the SSR Mg_2_Si come from the previous report.^24^

TE properties measurements were carried out on the fabricated pellets and legs after hot-press sintering, ensuring high densities above 95% of theoretical values calculated by Rietveld analysis (Table S1). However, the calculated and experimental density values of the SSR phase are lower than those of the MA counterpart due to the Sn losses. The deviation of stoichiometry from the nominal value and its shift closer to that of the Si end-member phase leads to a lower density calculated by Rietveld analysis, and this is also confirmed by the obtained experimental value. Figure 5 shows electrical conductivity and Seebeck coefficient data as a function of temperature for the developed materials as well as for the SSR-based Mg_2_Si of our previous study.^24^ As can be observed, there is a significant increase in electrical conductivity in the whole temperature range for both MA-based cases compared with those of SSR-based counterparts. Comparing the electrical conductivities of two MA-based samples, a notable increase is observed for the Si-end member phase. On the other hand, the two SSR cases demonstrate similar values across the whole *T* range. Seebeck coefficient data present negative values, characteristic of *n*-type materials. The two MA-based materials demonstrate a clear reduction of the Seebeck coefficient in absolute value compared with the SSR-based counterparts, indicating that MA-based materials exhibit higher levels of electron density. Moreover, comparing the MA-based materials, Mg_2_Si presents a small decrease in the Seebeck coefficient at higher temperatures, which means slightly higher charge carrier levels as expected according to previous reports.^19,44,50^ The increased electron density justifies the improved electron conductivity of two MA-based materials in comparison with those of SSR-based compounds. Since the electron density seems to change slightly when comparing the two MA-based systems, the mobility may be improved greatly for the Si-end member, contributing strongly to the notably increased values of electrical conductivity. This suggestion sounds logical because there is no solid solution in the Si-end member, which implies less atomic imperfections due to mass fluctuations. Moreover, the homogeneous microstructure observed for the MA-based Mg_2_Si implies less complexity and possibly an improved carrier mobility compared with that of the Mg_2_Si_0.8_Sn_0.2_ solid solution. The results from EDS elemental analysis reveal that the SSR-based Mg_2_Si_0.8_Sn_0.2_ presents deficient Bi doping. Only 0.015 of Si is substituted by Bi, providing almost the half of the total carrier number which seems to occur for the MA-based case. This confirms the suggestion for a poor electron density and explains the notable changes in the electrical transport properties in Figure 5. The losses of Bi and Sn in the SSR case may be caused during the hot-press sintering due to the low melting points of the two elements, while Sn losses have also been observed in previous reports.^18^ Moreover, the almost zero Mg_*i*_ content that is revealed for the SSR case by Rietveld analysis is another critical parameter for the electron doping deficiency and the low levels of electron density since practically all Mg excess is lost during the heat treatment process. As discussed previously, Mg losses are a quite common phenomenon during heating treatments and hot-press sintering. The heat treatment at 600 °C under vacuum caused more severe Mg losses compared to those that took place in the MA method, where every 5 min a pausing was set up to cool down the temperature during ball milling.

**Figure 6:**
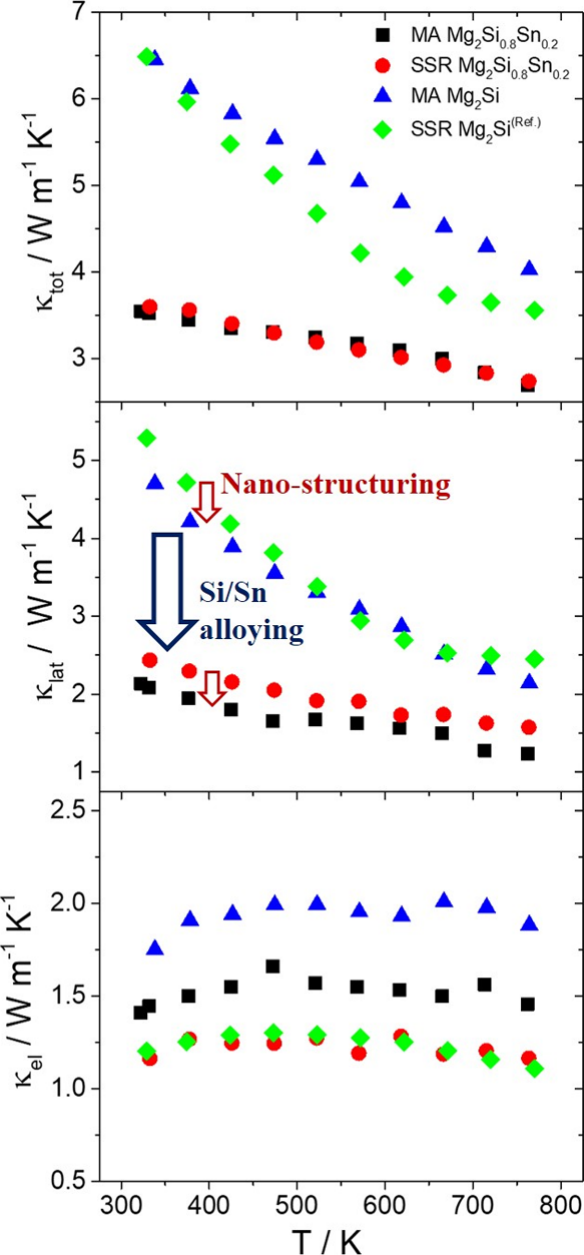
Total (κ_tot_), lattice (κ_lat_), and electronic (κ_el_) thermal conductivity as a function of temperature for Bi-doped Mg_2_Si_0.8_Sn_0.2_ and Mg_2_Si developed by SSR and MA. Data for the SSR Mg_2_Si come from the previous report.^24^

Figure 6 shows total (κ_tot_) thermal conductivity measurements as well as lattice (κ_lat_) and electronic (κ_el_) contributions as a function of the temperature. The electronic contribution was estimated using the Wiedemann–Franz law: κ_el_ = *L*·σ·Τ. The Lorenz number (*L*) was calculated by using Fermi–Dirac statistics and the Seebeck coefficient data considering scattering from acoustic phonons.^18,24^ Subtracting the electronic contribution from the total thermal conductivity (κ_tot_–κ_el_) the lattice thermal conductivity was determined. As observed, there is a pronounced difference in thermal conductivity values for the two systems. The high values of Mg_2_Si phases come clearly from the increased lattice contribution. Atomic imperfections through mass fluctuations caused by the heavier Sn atoms are absent in the homogeneous structure of Mg_2_Si anymore, and as a result, the scattering of phonons weakens markedly compared with the Si/Sn alloying cases. A noticeable reduction in lattice contribution is also observed for the MA-based samples in comparison with their SSR counterparts. As discussed previously, this decrease seems to come from the nanostructuring effect of ball milling process which decreases the grain size and increases the number of grain boundaries, enhancing the scattering of phonons with medium and long mean free path. On the other hand, as expected, the electronic thermal conductivity follows the trend of electrical conductivity. Its contribution is more pronounced in the case of MA-based Mg_2_Si which exhibits the highest electrical conductivity values. This is the reason that this material exhibits higher thermal conductivity values compared with its SSR counterpart. For the solid solution system, the total thermal conductivities of the SSR and MA phases reach almost the same values. The increase of electronic contribution, due the increase of electron density, counterbalances the reduction in lattice thermal conductivity, due to the nanostructuring effect in the MA-based Mg_2_Si_0.8_Sn_0.2_. As a result, the total thermal conductivity is at the same level with that of the SSR case.

**Figure 7:**
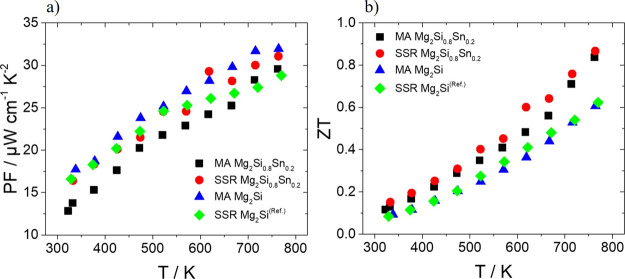
(a) PF and (b) *ZT* as a function of temperature for Bi-doped Mg_2_Si_0.8_Sn_0.2_ and Mg_2_Si developed by SSR and MA. Data for the SSR Mg_2_Si come from the previous report.^24^

Figure 7 shows PF and *ZT* data determined by previous physical property measurements. As observed, there is a notable difference in the PF between the two Mg_2_Si phases at higher temperatures. The highest values are exhibited by the MA-based material due to its higher doping level and electrical conductivity. At 773 K, this material demonstrates one of the highest PF of 32 μW cm^–1^ K^–2^ ever reported for the Si-end member phase which is comparable with the best values of Sn-rich members from this material group.^18,22,31^ On the other hand, in the Mg_2_Si_0.8_Sn_0.2_ system, there are no great differences in PF apart from a slight increase observed for the SSR case. Here, the low electrical conductivity of the SSR phase is counterbalanced by the small increase in the Seebeck coefficient, while in the MA system, the electrical conductivity and Seebeck coefficient follow the opposite trend due to the enhanced electron density. For this reason, together with the same levels of thermal conductivity, the SSR- and MA-based phases present similar *ZT* values. Comparing now the performance of the two systems, higher *ZT* values are exhibited by the two Si/Sn solid solution cases than those of the two Si-end member phases, with a more pronounced increase at higher temperatures. Obviously, the low levels of thermal conductivity and specifically of the lattice contribution significantly enhance the TE performance of the Sn-based compounds. Both MA- and SSR-based Mg_2_Si_0.8_Sn_0.2_ compounds exhibit a similar *ZT* of *ca*. 0.85 at 773 K, while the binary phases reach a *ZT* close to 0.6.

### Si Kerf-Based Mg_2_Si_0.8_Sn_0.2_ and Mg_2_Si Developed by MA

In an effort to utilize more effectively recyclable Si, Si-rich phases, Mg_2_Si_0.8_Sn_0.2_ and Mg_2_Si, were developed by MA using two types of Si kerf coming from the PV industry with the codes RST 1–2 and RST ODIN-0821, respectively. The MA method was chosen instead of SSR because the previous results showed that MA-based compounds present a higher accuracy and control of stoichiometric composition in terms of Mg and Sn losses as well as a more efficient electron doping through Bi substitution. Moreover, MA is a straightforward and scalable method, while in parallel the MA-developed TE silicide materials exhibit similar *ZT* values compared with those of SSR-based ones as shown in this study and our previous report.^18^ The kerfs originate from the silicon ingot slicing process during PV manufacturing. The two types of kerfs were processed and purified by different methods. Both types of kerf present relatively good purities with percentages more than 98.5% with the RST1–2 exhibiting slightly higher purity levels. Elemental analysis was carried out, and traces of elements such as Al, Ca, and Ni were identified in the scale of ppm, while a thin oxide layer was also detected on the surface of Si kerf nanoparticles by using scanning transmission electron microscopy with electron energy loss spectroscopy (STEM-EELS). Detailed characterization results are reported in our previous study.^44^

The two Si kerf-based systems were developed by applying the same MA and hot-press sintering conditions as previously. Powder XRD Rietveld refinements confirm the desired antifluorite-silicide phase for both Mg_2_Si_0.8_Sn_0.2_ and Mg_2_Si compounds with good agreement of experimental and calculated profiles (5.7 ≤ *R*_wp_/% ≤ 9.0). Rietveld refinement profiles for all developed Si kerf-based phases are presented in the Supporting Information. The thermal parameters were constrained to be equivalent for all elements, while their occupancy factors were set to their nominal values as concluded previously for the MA Si-5N-based counterparts. The occupancy of Mg_*i*_ atoms at the central 4*b* site was also investigated for all kerf-based cases. The refined crystallographic parameters are listed in Table 3.

**Table 3:** Refined Parameters from Rietveld Analysis of Powder XRD Data for Bi-Doped Mg_2_Si_1–*x*_Sn_*x*_, (*x* = 0 and 0.2) Based on RST 1–2 and RST ODIN-0821 Si Kerfs

Si-rich phases	*a* [Å]	*V* [Å^3^]	Mg_*i*_^a^ [%]	*U*_iso_ [Å^2^]	MgO [wt %]	Sn-rich^b^ [wt %]	*R*_wp_ [%]
Mg_2_Si_0.8_Sn_0.2_							
RST 1–2	6.41660(3)	264.189(4)	8.9(3)	1.65(3)	8.4(1)	7.2(4)	6.3
RST ODIN-0821	6.41470(3)	263.955(4)	9.5(3)	1.82(3)	8.2(1)	3.5(2)	5.7
Mg_2_Si							
RST 1–2	6.35866(3)	257.097(4)	0.9(3)	1.72(4)	7.6(1)		8.7
RST ODIN-0821	6.35870(3)	257.102(4)	1.1(3)	1.93(4)	8.9(1)		9.0

a Occupancy of interstitial Mg at the crystallographic site 4*b* (1/2 1/2 1/2).

b Secondary silicide phase with Sn-rich content.

As expected, the lattice constant follows a similar reduction with the previous results of Si-5N MA-based counterparts, moving from the Si/Sn solid solution to the Si-end member, for both Si kerfs, causing a similar contraction of the unit cell. Interestingly, the percentages of Mg_*i*_ atoms are also at similar levels in the two systems compared with the Si-5N counterparts, confirming the abrupt reduction of Si-end member phases close to ∼1%. MgO is also detected for these materials which present higher percentages above 7.5 wt %, compared with those of the pure Si-5N-based counterparts. This is in agreement with our previous report which also showed increased percentages for the kerf-based Mg_2_Si_0.4_Sn_0.6_ and Mg_2_Si_0.6_Sn_0.4_ phases. This increase of MgO may be attributed to the thin silicon oxide layer as well as other impurities detected in the Si kerfs.^44^ In addition, a distinct new silicide phase from the main one at slightly lower angles, with Sn-rich content, is confirmed by Rietveld analysis for the two kerf-based Mg_2_Si_0.8_Sn_0.2_. Refinements were executed at the Si/Sn occupancy factors at the 4*a* site to estimate the content of these phases. For the RST 1–2-based case, the formation of Mg_2_Si_0.56(3)_Sn_0.44(3)_ is estimated with a percentage of *ca*. 7.1 wt %, while for the RST ODIN-0821 compound, the results show Mg_2_Si_0.25(4)_Sn_0.75(4)_ as a secondary phase with a percentage of *ca*. 3.5 wt %. The formation of Sn-rich/Si-poor secondary phases could be explained by the fact that the two Si kerfs present lower purity levels compared with the Si-5N and consequently have a slightly lower percent of pure Si (∼1 to 1.5%).^44^ The Si deficiency probably results in a slight Sn excess, which finally favors the formation of Sn-rich phases at low levels. The absence of Sn and Sn/Si solid solutions in the Si-end members results in a single silicide phase for the two kerf-based materials (apart from the unavoidable MgO).

Backscattering SEM imaging confirms the formation of multiple phases for the two kerf-based Mg_2_Si_0.8_Sn_0.2_, as also shown previously for the Si-5N counterpart (Supporting Information, Figure S7). As expected, the two kerf Si-end members present a homogeneous structure without indication of multiple phases. Results from EDS elementary analysis for the two kerf-based systems are shown in Table 4. The Si/Sn ratios of two kerf-based Mg_2_Si_0.8_Sn_0.2_ phases do not show notable deviations from the nominal value, being at the same levels as that of the pure Si-5N MA-based counterpart in Table 2. The RST ODIN-0821 case presents only slightly lower levels of Sn, denoting small losses. The Bi dopant content is also in good levels, relatively close to the nominal value, for both Mg_2_Si_0.8_Sn_0.2_ and Mg_2_Si systems. However, it must be noticed here that these percentages are slightly lower than those of Si-5N MA-based counterparts, which affect the electrical transport properties as shown below.

**Table 4:** Results from the EDS Elemental Analysis of the Bi-Doped Mg_2_Si_1–*x*_Sn_*x*_, (*x* = 0 and 0.2) Based on the RST 1–2 and RST ODIN-0821 Si Kerfs

Si-rich phases	Si	Sn	Bi
Mg_2_Si_0.8_Sn_0.2_			
nominal	0.770	0.200	0.030
RST 1–2	0.804(1)	0.171(2)	0.025(1)
RST ODIN-0821	0.822(3)	0.155(2)	0.023(1)
Mg_2_Si			
nominal	0.970		0.030
RST 1–2	0.977(1)		0.023(1)
RST ODIN-0821	0.978(1)		0.022(1)

**Figure 8:**
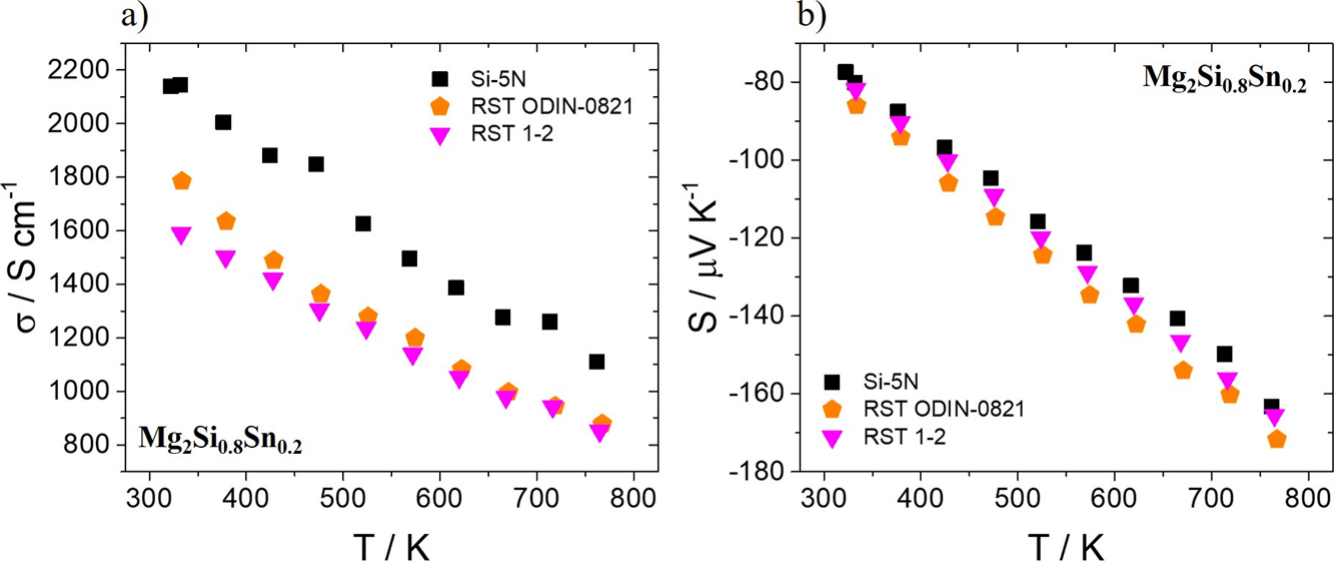
(a) Electrical conductivity and (b) Seebeck coefficient measurements as a function of temperature for Bi-doped Mg_2_Si_0.8_Sn_0.2_ based on Si-5N and two Si kerfs, RST ODIN-0821 and RST 1–2.

**Figure 9:**
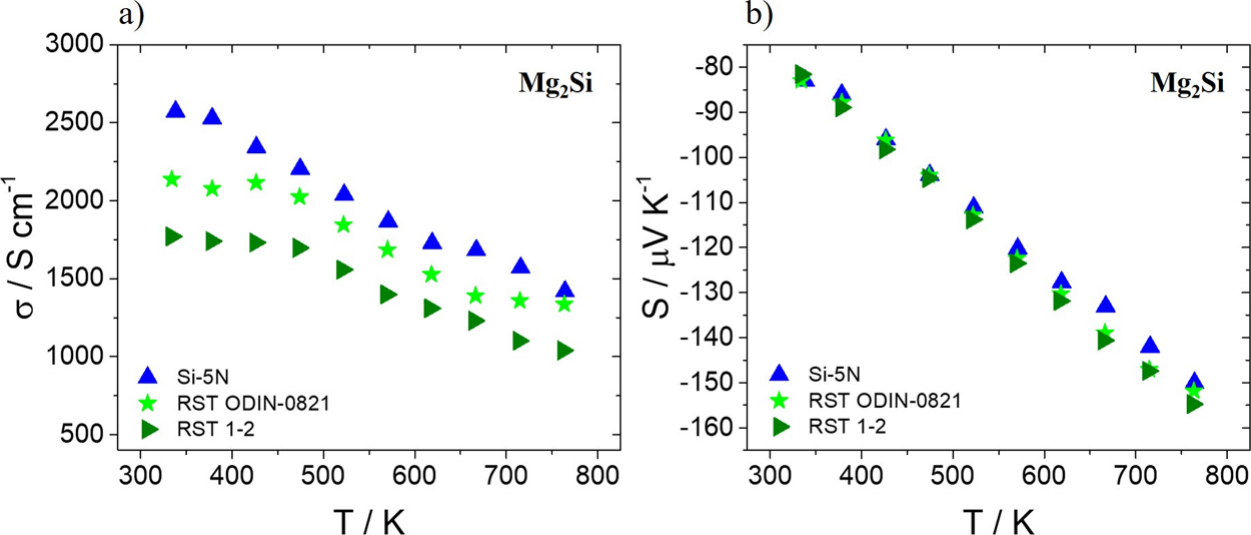
(a) Electrical conductivity and (b) Seebeck coefficient measurements as a function of temperature for Bi-doped Mg_2_Si based on Si-5N and two Si kerfs, RST ODIN-0821 and RST 1–2.

Figure 8 shows electrical conductivity and Seebeck coefficient measurements as a function of temperature for the two kerf-based Mg_2_Si_0.8_Sn_0.2_ phases in comparison with their Si-5N counterpart. As can be observed in Figure 8a, there is a similar reduction of electrical conductivity for the two kerf cases in comparison with that of the Si pure phase. In Figure 8b, the small increase of absolute Seebeck coefficient values for both *n*-type kerf-based materials indicates that their electron density is at lower levels in comparison to the Si-5N compound. The EDS results revealed slightly lower percentages of Bi dopant for the two kerf cases, enhancing the suggestion that a deficient electron doping occurs with negative effects in the electron density of these materials. Therefore, it is concluded that possibly the pronounced decrease of electrical conductivity is partially affected by the decrease of electron density. In addition, the increased percentages of impurities such as MgO in both kerf-based phases may cause a deterioration of electron mobility, as also suggested in our previous report.^44^ The decrease in electron mobility in combination with the reduction in carrier density seems to be responsible for this notable electrical conductivity decline. Similar decreasing trends are observed in Figure 9 for the electrical conductivity and Seebeck coefficient of the kerf-based Mg_2_Si phases, which are also attributed to a light Bi doping deficiency according to EDS results and a deterioration of electron mobility due to increased percentages of MgO and other impurities derived by the two kerfs.

Comparing the electrical transport properties of two kerfs, it must be noted that the RST ODIN-0821 phases in both systems present higher electrical conductivity values with a more pronounced increase in the Mg_2_Si system. It is also notable that for both Mg_2_Si_0.8_Sn_0.2_ and Mg_2_Si systems, Rietveld refinements showed higher Mg_*i*_ percentages for the RST ODIN-0821 cases compared with those of the RST 1–2 counterparts. Similar trends were also observed in the previous report for Mg_2_Si_0.4_Sn_0.6_ and Mg_2_Si_0.6_Sn_0.4_.^44^ Previous studies have shown that Mg_*i*_ plays an important role in the improvement of electrical transport properties since it provides two electrons in the electronic structure and works in synergy with Bi doping, which donates one electron. The combination of EDS and Rietveld analysis reveals that the Mg_*i*_ contribution to electron doping is stronger than Bi substitution, and this may be the reason why the RST ODIN-0821 case exhibits an improved electrical conductivity. This is more obvious in the Mg_2_Si system where the contribution in electron doping from Bi content is almost at the same level for both kerfs according to EDS results (*ca*. 2.2(1) % for RST ODIN-0821 and *ca*. 2.3(1) % for RST 1–2). The slight increase in Mg_*i*_ content observed for RST ODIN-0821 by Rietveld analysis is possibly responsible for the increase in electron concentration and consequently the appreciable improvement of electrical conductivity. In the Mg_2_Si_0.8_Sn_0.2_ system, EDS analysis showed an increase of Bi content for RST 1–2 case of *ca*. 0.2%, while Rietveld refinements revealed a higher Mg_*i*_ percentage of *ca*. 0.6% for the RST ODIN-0821 case. As a result, comparing the overall electron doping contributions in the two kerf-based phases, the RST ODIN-0821 case also seems to exhibit higher levels in electron concentration. As shown in Figure 8a, this results in a small improvement in electrical conductivity, which is more obvious especially in the region close to room temperature.

**Figure 10:**
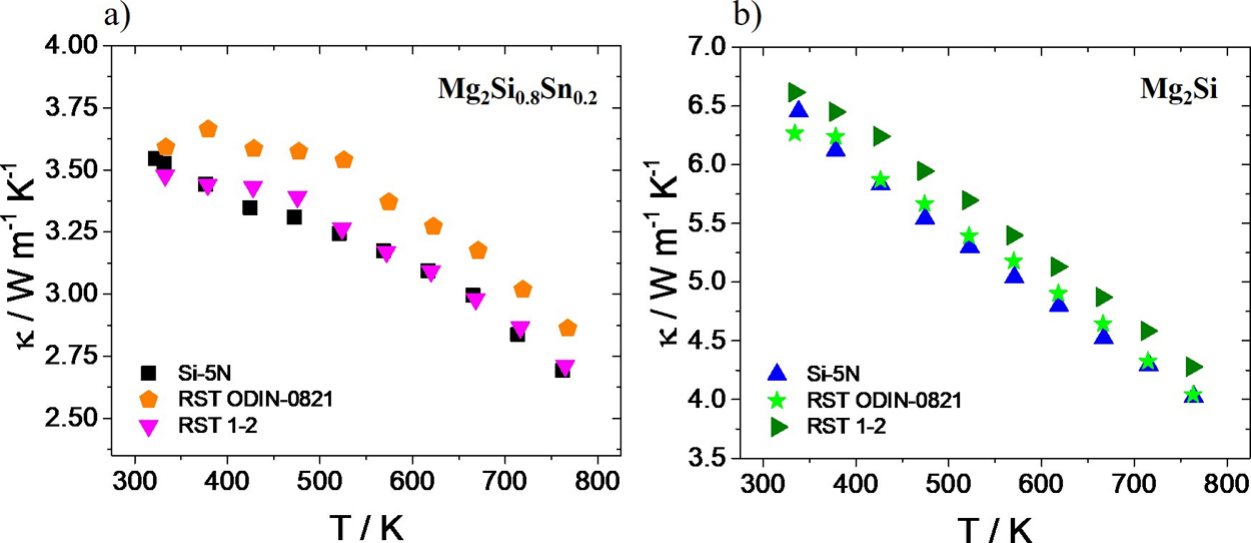
Thermal conductivity measurements as a function of temperature for (a) Bi-doped Mg_2_Si_0.8_Sn_0.2_ and (b) Bi-doped Mg_2_Si phases based on Si-5N and two Si kerfs, RST ODIN-0821 and RST 1–2.

**Figure 11:**
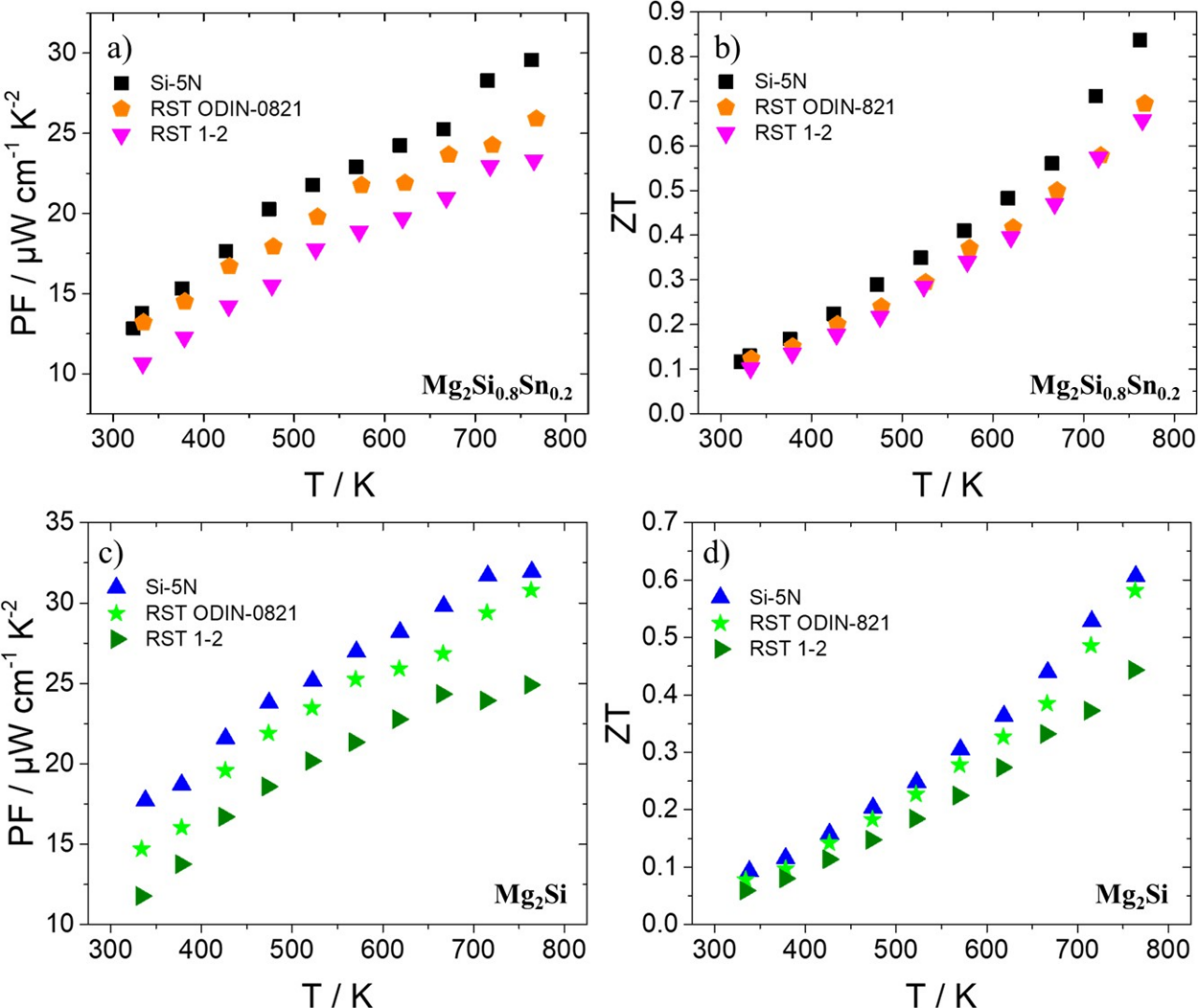
(a) PF and (b) *ZT* as a function of temperature for Bi-doped Mg_2_Si_0.8_Sn_0.2_ based on Si-5N and two Si kerfs, RST ODIN-0821 and RST 1–2. (c) PF and (d) *ZT* as a function of temperature for Bi-doped Mg_2_Si based on Si-5N and two Si kerfs, RST ODIN-0821 and RST 1–2.

Figure 10 presents thermal conductivity measurements as a function of temperature for Mg_2_Si_0.8_Sn_0.2_ and Mg_2_Si phases, respectively. In both systems, the kerf-based materials exhibit similar values compared with those of pure Si phases. Taking into account the aforementioned physical property measurement, the TE performance was determined for the kerf-based materials. Figure 11 shows PF and *ZT* data for the kerf-based Mg_2_Si_0.8_Sn_0.2_ and Mg_2_Si phases compared with their Si pure counterparts. As expected, all kerf-based phases present lower PF values in comparison with those of commercial Si cases mainly due to the reduction of electrical conductivity, since there are no great changes in the Seebeck coefficient. However, the RST ODIN-0821-based materials exhibit a higher PF than that of RST 1–2 counterparts across the whole temperature range, since they demonstrate electrical conductivity values closer to those of Si-5N phases. This is more pronounced for the RST ODIN-0821-based Mg_2_Si which exhibits a remarkable maximum PF of *ca*. 31 μW cm^–1^ K^–2^ which is quite close to that of the commercial Si case.

The TE *ZT* values of both systems follow the trends of PF since thermal conductivity measurements did not show notable differences between the kerf-based phases and their Si-5N counterparts. As a result, the RST ODIN-0821-based phases exhibit higher TE performance in both systems compared with their RST 1–2 counterparts. At 773 K the RST ODIN-0821-based Mg_2_Si_0.8_Sn_0.2_ and Mg_2_Si phases demonstrate maximum *ZT* values of 0.70 and 0.58, respectively. Confirming the results of our previous report,^44^ the developed kerf-based Si-rich silicide phases demonstrate promising TE properties for future investigations and the potential for integration in green-energy applications with the aim of establishing a circular economy approach in the Si industry. This study shows that high-purity commercial Si can be replaced successfully by recyclable Si kerf from PV manufacturing for the development of Si-rich silicide TE materials with promising efficiencies, following a straightforward and scalable synthesis method such as MA. As also confirmed in our previous study for Mg_2_Si_0.6_Sn_0.4_ phases,^18^ MA is more advantageous than the SSR method for the Si-rich systems, Mg_2_Si_0.8_Sn_0.2_ and Mg_2_Si. It is proven here that MA-developed silicide phases are doped more effectively by Bi substitution and exhibit higher accuracy in the stoichiometry of Mg, Bi, and Sn during the synthesis and hot-press sintering process. Therefore, MA provides a better control of electron doping, since the aforementioned results showed a good consistency of real Bi content with the nominal values. In addition, through MA less amounts of Mg losses are observed, and therefore, more Mg is available to become Mg_*i*_, providing more efficiently an alternative route for electron doping and optimization of electrical transport properties.

## Conclusions

In conclusion, a straightforward and simple MA method is implemented for the first time to develop Bi-doped Si-rich silicide phases, Mg_2_Si_0.8_Sn_0.2_ and Mg_2_Si, while in parallel a comparative study of synthetic routes is also performed synthesizing Mg_2_Si_0.8_Sn_0.2_ by the SSR method. MA is proven to be an advantageous technique for the development of Si-rich phases compared with the SSR method. MA provides higher control and precision of produced stoichiometric compositions as well as more effective electron doping, since this method presents less elemental losses and deviations from nominal stoichiometric values, according to the results derived by three characterization techniques: EDS, powder XRD Rietveld refinements, and SEM analysis.

Powder XRD confirmed the desired antifluorite cubic structure for all developed materials, while slight amounts of the common secondary phase, MgO, were also identified. Rietveld analysis revealed remarkable Sn losses for the SSR-based Mg_2_Si_0.8_Sn_0.2_ at the 4*a* site, while the loss of excess Mg during the heat treatment results in a deficiency of interstitial Mg atoms at the 4*b* site. In contrast, the MA-based Mg_2_Si_0.8_Sn_0.2_ did not show notable changes from the Sn nominal value, presenting in parallel an appreciable Mg_*i*_ content at the 4*b* site. EDS confirmed significant Sn losses for the SSR case, together with a pronounced decrease in Bi content, while for the two MA-based phases, the elemental analysis showed a good agreement with the nominal values. Backscattering SEM imaging revealed a multiple phase microstructure with Sn-rich regions in the Si-rich matrix for both MA- and SSR-developed Mg_2_Si_0.8_Sn_0.2_ phases, while the Si end-member phase presents a homogeneous microstructure. Another important conclusion that may be correlated with the microstructure of investigated compounds and affects the electron transport properties is the percentage of Mg_*i*_ atoms. As Rietveld refinements showed for the MA cases, the notable differences in interstitial Mg content observed between the Mg_2_(Si,Sn) compounds and the binary phase may be connected with the Sn-rich regions in the solid solution cases, which probably present wider structures locally, and this favors the occupancy of Mg_*i*_ atoms at the central 4*b* site. Finally, SEM imaging revealed a porous microstructure for the SSR case which is indicative of Mg_2_(Sn,Bi) losses, as previous studies have shown, also validating the results from EDS and Rietveld analysis. Therefore, it is clearly obvious that the MA-based solid solution exhibits a more precise and controlled stoichiometry, closer to the nominal value, compared with its SSR counterpart, which presents pronounced Sn, Mg, and Bi losses. This has a strong impact on the electrical transport properties of materials, attributed to the electron doping deficiency of the SSR-phase. In addition, a reduction in lattice thermal conductivity is observed for the two MA-phases which is attributed to the nanostructuring effect of the ball milling process. Backscattering SEM imaging supports this suggestion since the number of smaller aggregates is increased for the MA solid solution case. For the aforementioned reasons, MA was preferred for the development of Mg_2_Si_0.8_Sn_0.2_ and Mg_2_Si phases by using two types of recyclable Si kerf with codes RST 1–2 and RST ODIN-0821 from PV manufacturing. The kerf-based compounds exhibit reduced electrical conductivities in comparison with their pure Si-5N counterparts, mostly due to the slight decreases of Bi content, as EDS confirmed, while the increased percentages of MgO in the kerf-based products may also deteriorate their electron mobility. However, the RST ODIN-0821-based phases exhibit better TE properties compared to those of RST 1–2 cases, with PF and *ZT* values closer to those of their Si-5N counterparts. At 773 K, a maximum *ZT* of 0.85 and 0.6 is reached by the Si-5N Mg_2_Si_0.8_Sn_0.2_ and Mg_2_Si phases, while their RST ODIN-0821-based counterparts exhibit a *ZT* of 0.70 and 0.58, respectively.

## Supporting Information Available

Powder XRD Rietveld refinement profiles presented for the remaining Βi-doped Mg_2_Si_0.8_Sn_0.2_ and Mg_2_Si compounds; backscattering SEM imaging illustrates the microstructure of the kerf-based Mg_2_Si_0.8_Sn_0.2_ and Mg_2_Si phases; and the experimental densities of fabricated pellets and their theoretical values calculated by Rietveld analysis (PDF)
