# Supplementary material for: Mechanical Alloying: An Advantageous Method for the Development of Mg2Si0.8Sn0.2 and Mg2Si Thermoelectrics Using Commercial and Recyclable Silicon
Source: ACS Appl Energy Mater. 2025 Jan 22;8(3):1783–95. doi: 10.1021/acsaem.4c03000 (PMC11815622; doi:10.1021/acsaem.4c03000)
Supplement: Supplementary file 1 — ae4c03000_si_001.pdf [file ae4c03000_si_001.pdf]

Mechanical Alloying: An advantageous method for the development of $Mg_2Si_{0.8}Sn_{0.2}$ and Mg_2Si thermoelectrics using commercial and recyclable silicon.

Panagiotis Mangelis,[1,*] *Panagiotis S. Ioannou,*[1] *Anne-Karin Søiland,*[2] *Theodora Kyratsi*[1,*]

[1]*Department of Mechanical and Manufacturing Engineering, University of Cyprus, 1678 Nicosia, Cyprus*

[2]*ReSiTec AS, Setesdalsveien 110, 4617 Kristiansand, Norway*

*mangelis.panagiotis@ucy.ac.cy, kyratsi@ucy.ac.cy

KEYWORDS: magnesium silicide, mechanical alloying, solid-state reaction, hot-press sintering, recycled silicon, thermoelectric properties

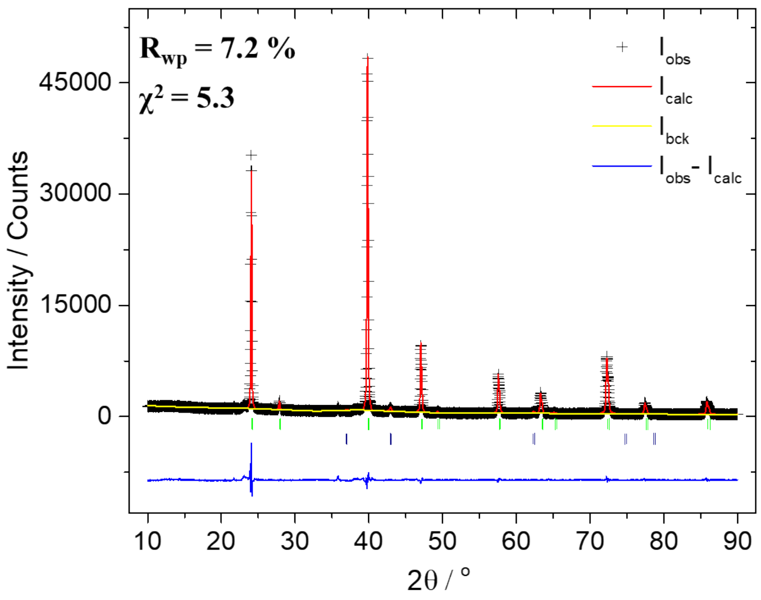

Figure S1 Powder XRD Rietveld refinement profile for the SSR-based $Mg_2Si_{0.8}Sn_{0.2}$: final observed (black crosses), calculated (red solid line), calculated background (yellow line) and difference (blue line). Reflection positions for the silicide phase are marked with green and for MgO with navy color.

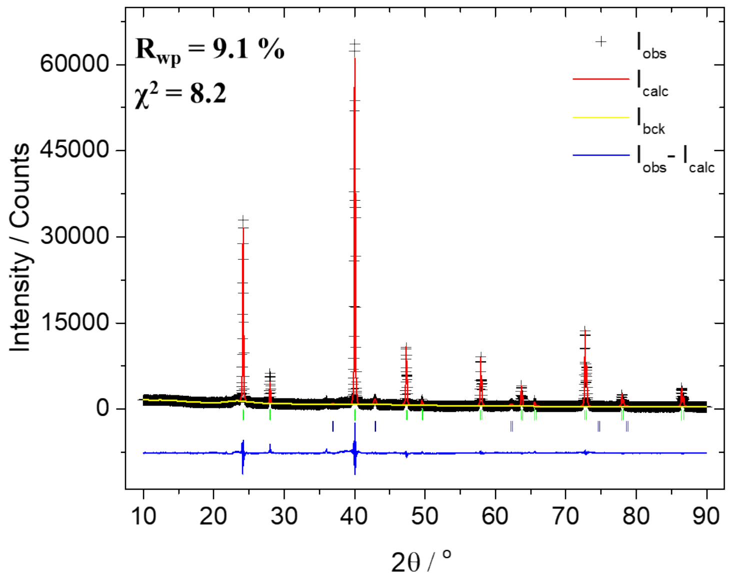

Figure S2 Powder XRD Rietveld refinement profile for the MA-based Mg_2Si using Si-5N: final observed (black crosses), calculated (red solid line), calculated background (yellow line) and difference (blue line). Reflection positions for the silicide phase are marked with green and for MgO with navy color.

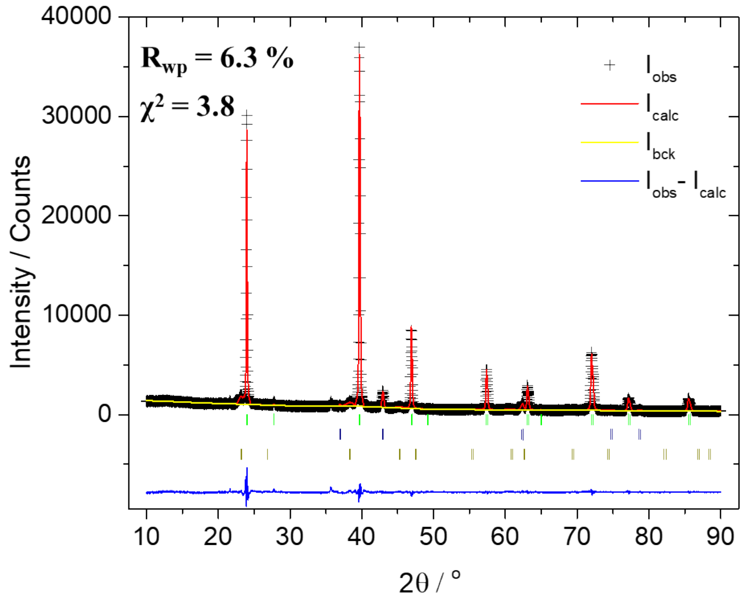

Figure S3 Powder XRD Rietveld refinement profile for the $Mg_2Si_{0.8}Sn_{0.2}$ using RST 1-2 kerf: final observed (black crosses), calculated (red solid line), calculated background (yellow line) and difference (blue line). Reflection positions for the silicide phase are marked with green, for MgO with navy and for the Sn-rich phase, $Mg_2Si_{0.56(3)}Sn_{0.44(3)}$, with dark yellow color.

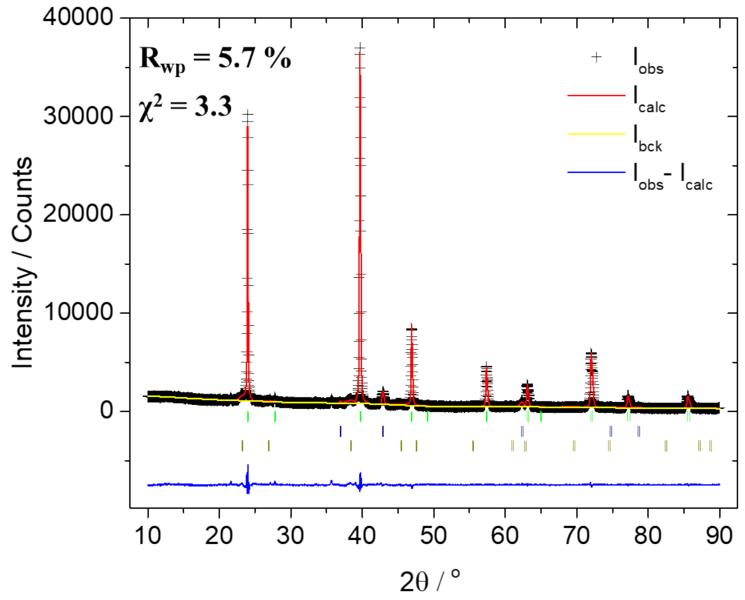

Figure S4 Powder XRD Rietveld refinement profile for the $Mg_2Si_{0.8}Sn_{0.2}$ using RST ODIN-0821 kerf: final observed (black crosses), calculated (red solid line), calculated background (yellow line) and difference (blue line). Reflection positions for the silicide phase are marked with green, for MgO with navy and for the Sn-rich phase, $Mg_2Si_{0.25(4)}Sn_{0.75(4)}$, with dark yellow color.

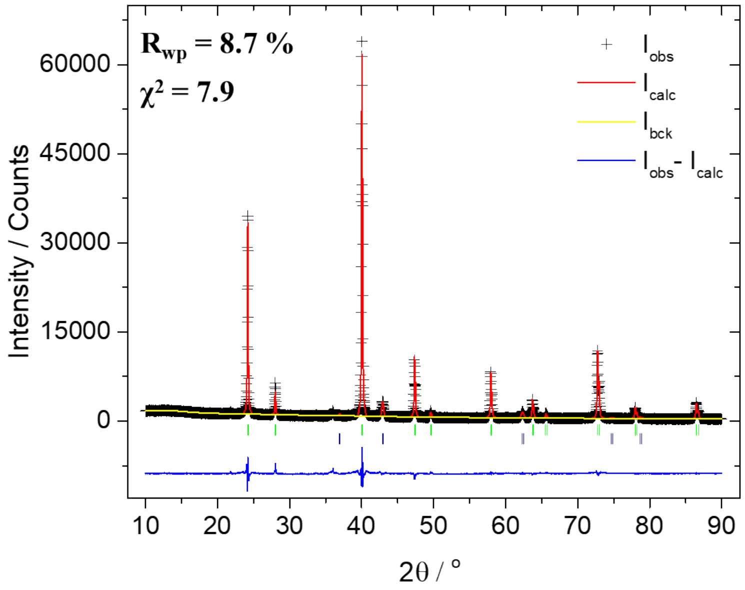

Figure S5 Powder XRD Rietveld refinement profile for the Mg_2Si using RST 1-2 kerf: final observed (black crosses), calculated (red solid line), calculated background (yellow line) and difference (blue line). Reflection positions for the silicide phase are marked with green and for MgO with navy color.

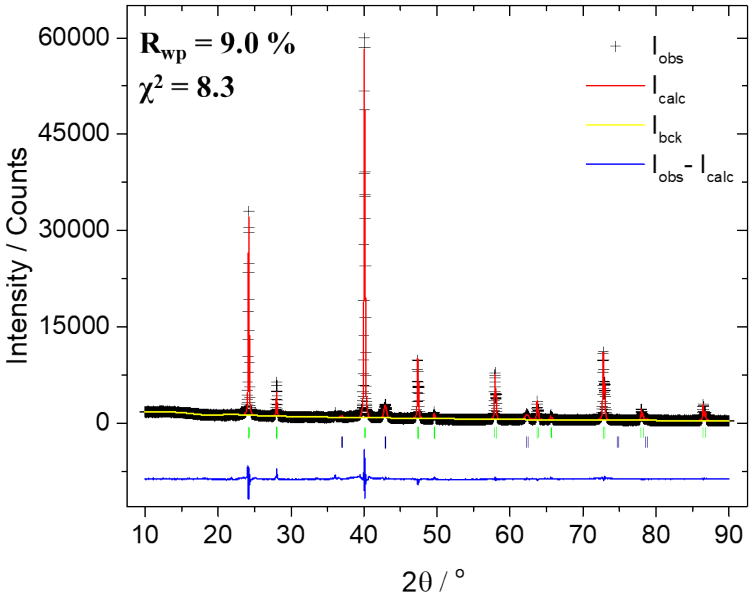

Figure S6 Powder XRD Rietveld refinement profile for the Mg_2Si using RST ODIN-0821 kerf: final observed (black crosses), calculated (red solid line), calculated background (yellow line) and difference (blue line). Reflection positions for the silicide phase are marked with green and for MgO with navy color.

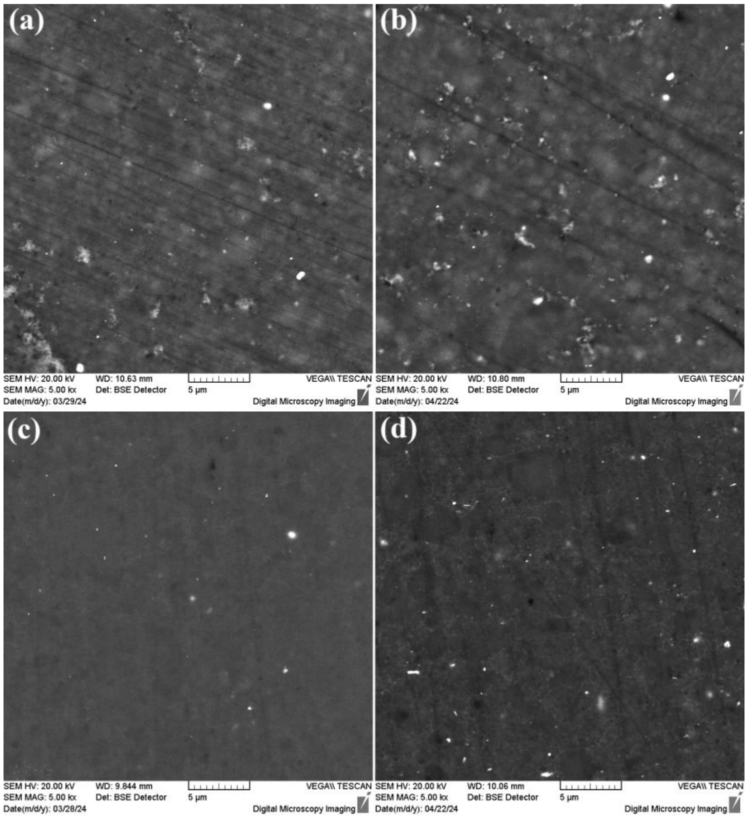

Figure S7 Backscattering SEM imaging for the a) RST 1-2-based $Mg_2Si_{0.8}Sn_{0.2}$ b) RST ODIN-0821-based $Mg_2Si_{0.8}Sn_{0.2}$ c) RST 1-2-based Mg_2Si and d) RST ODIN-0821-based Mg_2Si. The scale bar is 5 µm.

Table S1 Densities of fabricated $Mg_2Si_{1-x}Sn_x$ (x = 0, 0.2) pellets: experimental and theoretical values calculated by Rietveld analysis.

Si-rich Phases	Experimental / g cm^{-3}	Theoretical / g cm^{-3}
$Mg_2Si_{0.8}Sn_{0.2}$		
SSR, Si-5N	2.23	2.26
MA, Si-5N	2.45	2.56
RST 1-2	2.45	2.57
RST ODIN-0821	2.46	2.58
Mg_2Si		
MA, Si-5N	2.06	2.13
RST 1-2	2.05	2.13
RST ODIN-0821	2.11	2.13
